# A Simple, Rapid, and Practical Method for Distinguishing *Lonicerae Japonicae* Flos from *Lonicerae* Flos

**DOI:** 10.3390/molecules24193455

**Published:** 2019-09-23

**Authors:** Fang Zhang, Pengliang Shi, Hongyan Liu, Yongqing Zhang, Xiao Yu, Jing Li, Gaobin Pu

**Affiliations:** 1 College of Pharmacy, Shandong University of Traditional Chinese medicine, Jinan 250355, China; zfang_819@163.com (F.Z.); lhyan0@163.com (H.L.); ljsdnu@163.com (J.L.); gbpu@163.com (G.P.); 2 Shandong Medicine Technician College, Taian 271016, China; SPL_CN@163.com

**Keywords:** *Lonicerae japonicae* flos, *Lonicerae* flos, secologanic acid, high performance liquid chromatography, multiple statistical analysis

## Abstract

*Lonicerae japonicae* flos (LJF), the dried flower buds of *Lonicera japonica* Thunb., are often adulterated with *Lonicerae.* flos (LF), which is derived from the other four *Lonicera* species. Scholars at home and abroad have established several analytical methods to distinguish LJF from the four *Lonicera* species of LF; however, to date, no effective and practical method has been established for distinguishing LF from LJF. In our present study, the HPLC fingerprints of LJF and LF were compared, and differences in the content of one of the iridoids were found. Column chromatography combined with pre-HPLC was used for isolating and preparing the iridoid, and its structure was identified as secologanic acid. Then, a method for determining the content of secologanic acid was established using HPLC. The amounts of secologanic acid in 34 batches of LJF and 38 batches of LF were determined. The average amount of secologanic acid in 34 batches of LJF was 18.24 mg/g, with values ranging from 12.9 mg/g to 23.3 mg/g, whereas the average amount in 38 batches of LF was 1.76 mg/g, with values ranging from 0.2 mg/g to 7.2 mg/g. Therefore, secologanic acid can be considered as one of the characteristic components for distinguishing LJF and LF. Our study not only provides a rapid, simple, sensitive, and practical method for identifying LJF and LF but also establishes a method for discovering the characteristic components of other herb-medicines that are susceptible to adulteration.

## 1. Introduction

In global herbal markets, some substitutes or adulterants are often traded due to their relatively low costs [1,2]; species substitution may adversely affect consumer health, as it could cause severe allergies or not have the intended effect [3,4]. Visual detection of species adulteration in the raw herbal trade is often difficult, as the plants are usually in a dry state and do not retain the original features of the plant [5]. Therefore, accurate and rapid authentication of herbal raw materials is crucial for ensuring the safety and efficacy of medicinal or edible herbs and their preparation.

*Lonicerae japonicae* flos (LJF), the dried flower buds of *Lonicera japonica* Thunb., also called Japanese honeysuckle or jīn yín huā, possesses the functions of clearing heat, removing toxins, and dispersing wind-heat, which has been widely used in traditional Chinese medicine to treat various conditions, such as cough, fever, sore throat, and influenza infection [6]. In addition to a wide application in clinical Chinese medicine prescriptions, LJF is also a major component of various traditional Chinese medicine preparations, such as Shuanghuanglian tablets and oral liquid. More than 500 preparations containing LJF have been used to treat various diseases in China [7,8,9]. At the same time, it can also be used in functional foods, cosmetics, and other applications [10].

However, LJF is often substituted or adulterated with a related herbal medicine, *Lonicerae* flos (LF), also known as Shanyinhua [11]. According to the current 2015 edition of *Chinese Pharmacopoeia* (ChP) [12], LF can be the dried flower buds of any of the other four *Lonicera* species: *Lonicera macranthoides* Hand.-Mazz., *Lonicera hypoglauca* Miq., *Lonicera confusa* DC., or *Lonicera fulvotomentosa* Hsu et S. C. Cheng. Because of the high yields and low prices, LF is often traded as LJF in herbal markets. These two kinds of herb-medicine are so similar in morphological properties that it is very difficult to distinguish them with the naked eye.

To distinguish LJF from the four *Lonicera* species of LF, scholars at home and abroad have established such analytical methods as morphological and microscopic identification, spectral identification, physicochemical identification, and biotechnological identification. For example, stereo microscopy and optical microscopy were used to identify LJF and LF, and differences in the calyx tube, corolla hairs, and glandular hairs were found [13]. Proton nuclear magnetic resonance (^1^H-NMR) fingerprints were applied and the results showed that the differences were mainly reflected in the high field region (δ 0.5–3.0) and the sugar terminal substrates region (δ 3.0–5.5) [14]. The single nucleotide polymorphism (SNP) double peak technique method was also used to distinguish LF adulteration phenomena, and the results showed that only 17% of botanical extracts and 22% of LJF Chinese patent medicines did not contain adulteration [15]. These analytical methods can achieve the goal of identifying LJF and LF to some extent, but there are still limitations. For example, microscopic identification was limited to small differences in identification points and poor practicability, and the ^1^H-NMR fingerprints and DNA barcoding technology had the disadvantages of being expensive and complex, which made them difficult to popularize.

**Figure 1:**
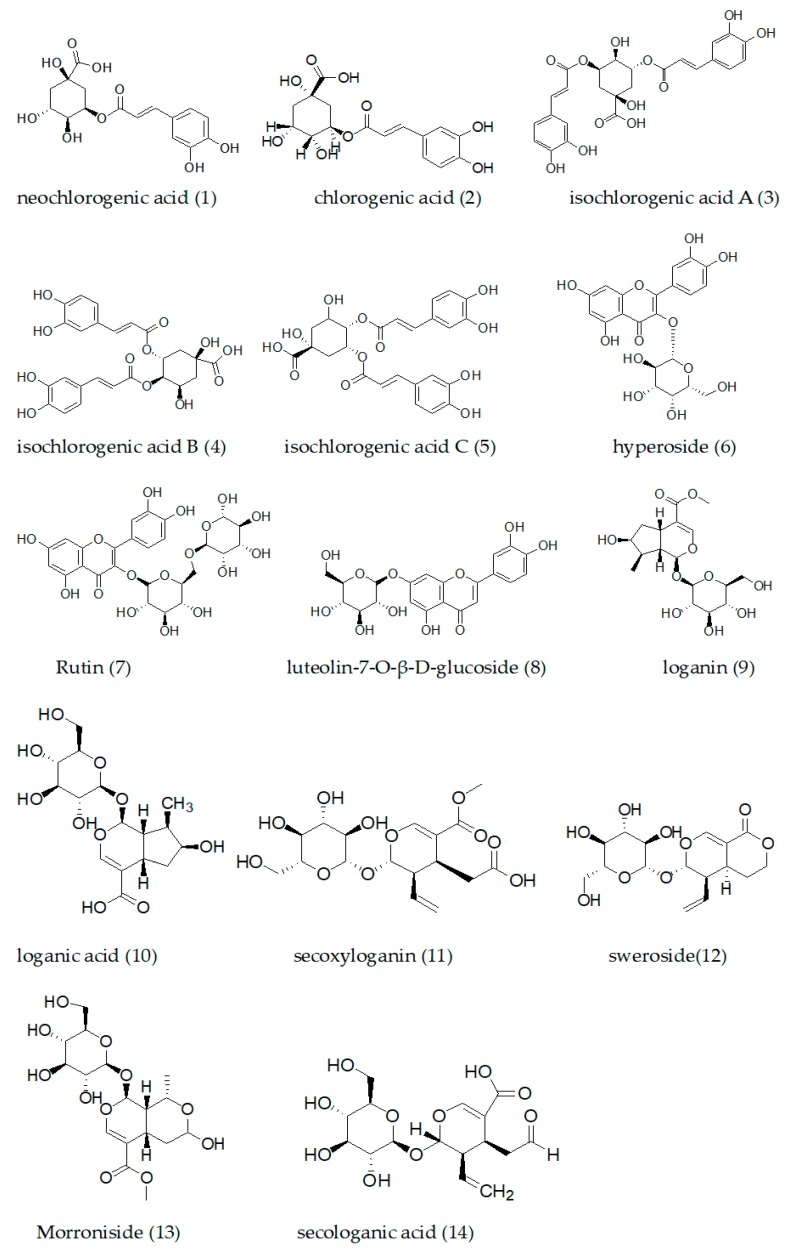
Chemical structures of the representative components in flower buds of *Lonicerae* species (1–14).

Previous research indicated that the flower buds of *Lonicera* species contain a variety of compounds, such as phenolic acids, flavonoids, triterpenoid saponins, and iridoids (Figure 1) [8]. Because of the similarity of the chemical components, it is difficult to effectively distinguish LJF from LF by chemical qualitative analysis. However, when a further study was carried out on LJF and LF, the scholars found that LF contained abundant saponins, but LJF did not, and LJF had more flavonoids and iridoids than LF [16]. Therefore, iridoids or saponins were taken as one of the indicators for distinguishing LJF from LF [17,18]. However, based on Gaoshan’s report, Macranthoidin B and Dipsacoside B in *L. macranthoides* and *L. hypoglauca* met the requirements of ChP (no less than 5.0%), but no Macranthoidin B and Dipsacoside B was detected in the samples of *L. confusa* [19]. Chen et al. reported that *L. japonica* was more abundant in iridoid glycosides, including sweroside, 7-epi-vogeloside, centauroside, and loganin, than LF [16], yet Zhang et al. found sweroside in LJF and three species of LF, and the amount of sweroside in *L. macranthoides* was basically the same as that in LJF [20]. Therefore, the adaptability of taking saponins or iridoids as indexes to distinguish LF from LJF required further study. As discussed above, no effective and practical method has been established for distinguishing LF from LJF until now.

In our present study, the HPLC fingerprints of LJF and LF were matched automatically by the chromatographic fingerprint of Traditional Chinese Medicine (TCM) (Version 2004A), and significant differences, especially in the content of some iridoids, were found. Column chromatography combined with pre-HPLC was used for isolating and preparing the iridoid, and its structure was identified as secologanic acid. Then, a method for determining the amount of secologanic acid was also established using HPLC.

## 2. Results

### 2.1. A Comparison of Fingerprints between LF and LJF

**Figure 2:**
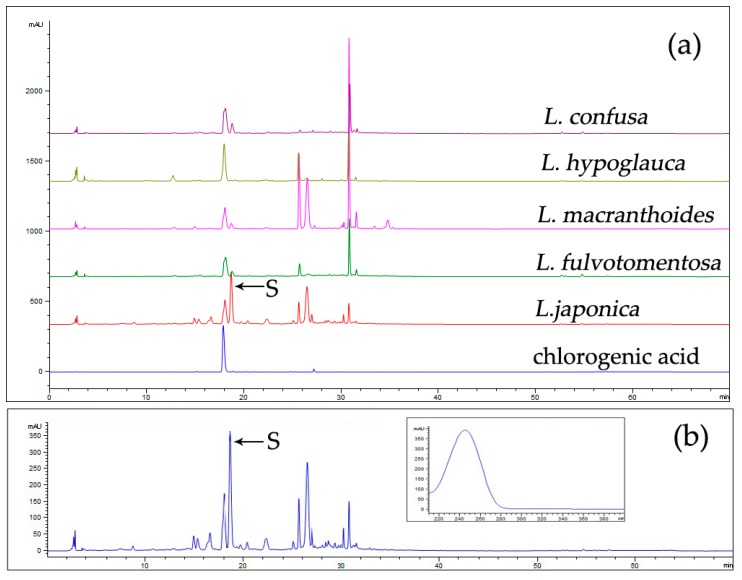
The overlapping fingerprints of *L. japonicae* flos (LJF) and LF (**a**) and the UV spectrum of compound S in LJF (**b**). Column: An Agilent ZORBAX XDB C18 (4.6 × 250 mm, 5 μm) column; column temperature, 30 °C; mobile phases: phosphoric acid solution (A) and acetonitrile (B) (5–15% B at 0–20 min, 15–55% B at 20–45 min, and 55–100% B at 45–60 min); flow rate: 1.0 mL/min; detection wavelength: 254 nm; injection volume: 10 µL.

The overlapping fingerprints of LJF and LF are shown in Figure 2a, with chlorogenic acid as a reference. The differences between them are mainly shown in the presence or absence of some peaks, especially peak S. Under the same extraction conditions, including sample weight, solvent volume, and extraction method, and the same chromatographic conditions, the peak area of secologanic acid from LJF was 5373.4, whereas those of LF were 580.6 (*L. macranthoides*), 0.0 (*L. hypoglauca*), 1052.8 *(L. confusa)*, and 597.1 (*L. fulvotomentosa*). Therefore, the peak area of compound S in LJF was significantly higher than in LF, and it can be inferred that LJF is rich in component S, whereas LF is lower.

The UV absorption spectrum (Figure 2b) of peak S at the full wavelength shows that it was consistent with the absorption spectrum of iridoids, so the peak was identified as one of the iridoids.

### 2.2. Preparation of Crude Extract for Separation of Compound S

**Figure 3:**
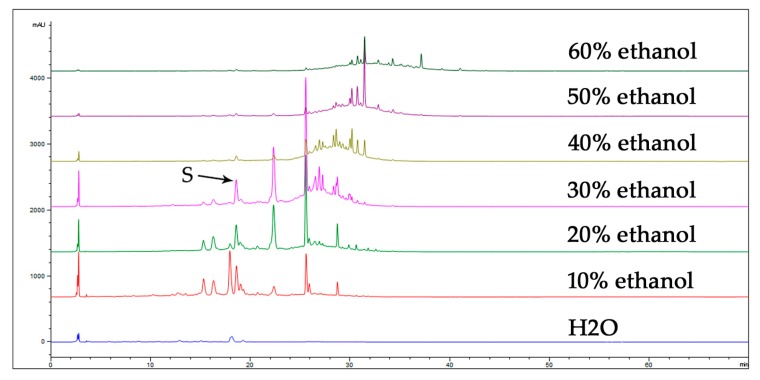
HPLC chromatograph of eluents eluted with H_2_O and ethanol of different concentrations on a macroporous resin. The chromatographic conditions were the same as those described in the HPLC fingerprint.

The HPLC results of the eluents achieved by eluting with H_2_O and ethanol at different concentrations on a D101 macroporous resin column is shown in Figure 3, and the compound S was mainly contained in eluents of 10%, 20%, and 30% ethanol. However, the impurity interference of neighboring peaks on both sides of compound S in the 30% ethanol eluent was less. Therefore, 30% ethanol eluent was chosen as the sample for preparing compound S in subsequent experiments.

### 2.3. The Separation, Purification, and Identification of compound S

**Figure 4:**
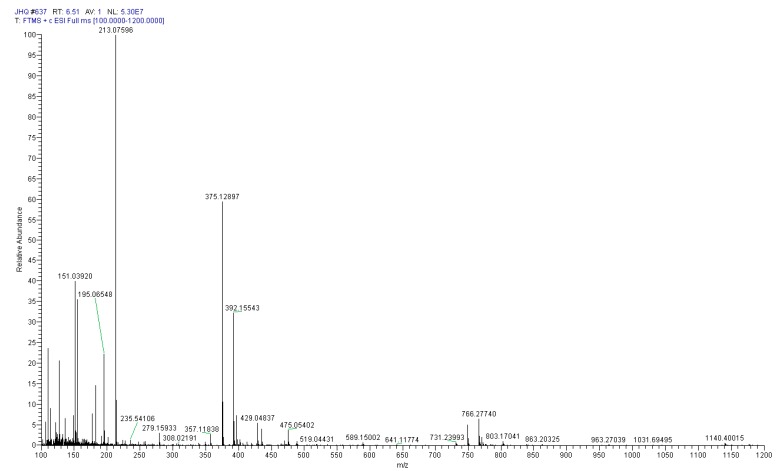
The Q Exactive Hybrid Quadrupole-Orbitrap-MS spectrum of compound S in positive mode.

After further separation and purification by a Sephardex LH-20 column and pre-HPLC, the purity of compound S was calculated by the peak area normalization method, and its purity was over 90%. Figure 4 displays the positive Q Exactive Hybrid Quadrupole-Orbitrap-MS spectrum of compound S, which shows an [M + H]^+^ ion at *m/z* 375.12897. The neutral loss of a glucose unit (162Da) generated the aglycone ion [M + H − Glc]^+^ at *m/z* 213.07596, and the loss of H_2_O from the aglycone ion produced the ion [M + H − Glc − H_2_O]^+^ at *m/z* 195.06548. The loss of C_2_H_4_O (44Da) generated the product ion [M + H – Glc − H_2_O − H_2_O − C_2_H_2_]^+^ at *m/z* 151.03920. The cracking rule of compound S is consistent with the secologanic acid reported in previously published spectra [21,22], so we preliminarily speculated that compound S was a secologanic acid. After comparing the retention time of compound S with the secologanic acid standard, the compound S was unambiguously assigned as secologanic acid.

### 2.4. Stability of Secologanic Acid

A previous study showed that secologanic acid almost completely converted to epivogeloside or vogeloside in methanol, whereas it hardly converted in pure water [23]. To select a suitable solvent for sample preparation, the stability of secologanic acid in different solvents was further studied. The results showed that the relative standard deviation (RSD, %) of the peak areas of secologanic acid were 5.06% (H_2_O), 1.31% (20% ethanol), 3.48% (50% ethanol), and 3.63% (50% methanol), and the RSD (%) of the standard solution was 0.51% within 27 h. The results showed that the secologanic acid was stable when the sample was extracted with 20% ethanol.

### 2.5. Optimization of the Extraction Conditions

The extraction efficiency was tested by comparing the amount of secologanic acid in different extraction solvents, including water and ethanol of different concentrations. The results showed that the sample extracted with water had the largest amount (17.11 mg/g), whereas the amounts in samples extracted with 10%, 20%, 30%, and 50% ethanol were relatively small (14.80, 14.86, 14.30, and 14.34 mg/g, respectively). The samples extracted with 75% and 85% ethanol contained the lowest amounts (11.51 and 11.85 mg/g, respectively). Given that the aqueous solution was susceptible to bacteria on hot days, 20% ethanol was chosen as the extract solvent.

**Figure 5:**
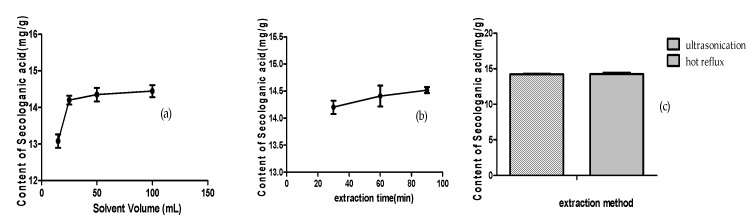
The content of secologanic acid in LJF under different extraction conditions: (**a**) solvent volume, (**b**) extraction time, (**c**) and extraction method.

The extraction conditions for LJF and LF were optimized with 20% ethanol as solvent by comparing different extraction methods (ultrasonication or hot reflux), the solvent volume (15, 25, 50, and 100 mL), and the extraction time (30, 60, and 90 min). The results are shown in Figure 5. Although the two extraction methods had almost the same efficiency, the ultrasonic extraction was selected, owing to its convenient operation and lower temperature, which could benefit the compound’s stability. When the samples were extracted with 25, 50, and 100 mL of 20% ethanol, there were no significant differences in the amounts of secologanic acid (14.20, 14.35, and 14.44 mg/g, respectively), whereas the sample extracted with 15 mL 20% ethanol contained the lowest amount (13.08 mg/g). The extraction time had no obvious effect on extraction efficiency, and the amounts of secologanin acid were 14.20 mg/g (30 min), 14.41 mg/g (60 min), and 14.52 mg/g (90 min), respectively. Therefore, the sample extraction conditions were optimized as follows: ultrasonication for 30 min with 25 mL of 20% ethanol.

### 2.6. The Optimization of Chromatographic Conditions

To obtain good separation results, the conditions of HPLC, including column, mobile phase, and column temperature, were examined. After comparing Agilent ZORBAX SB C_18_ (4.6 × 250 mm, 5 μm), Thermo Scientific Hypersil GOLD DimC_18_ (4.6 × 250 mm 5 μm), and Waters Symmetry C_18_ (4.6 × 250 mm, 5 μm), Agilent ZORBAX SB C_18_ column was selected for its excellent separating efficiency and peak shapes. In addition, varied mobile phases, such as acetonitrile/methanol with different concentrations of phosphoric acid (0.05%, 0.1%, and 0.2%, *v/v*), were tested. It was found that a mixture of acetonitrile and 0.1% (*v/v*) phosphoric acid was the most suitable mobile phase, which separated the secologanic acid with sharp peaks and lower background noise. Column temperature (25, 30, and 35 °C) was also examined, and 35 °C was appropriate for the analysis.

### 2.7. Linear and Analytical Method Validation

The calibration curves exhibited good linear regressions (r^2^ > 0.9999) during the investigated concentration range (2.264–5.66 µg/mL), and the calibration curve was A = 12.12X − 4. The limit of detection (LOD) and limit of qualification (LOQ) of the secologanic acid were 0.1415 and 0.566 µg/mL, respectively. The precision was measured based on intra- and inter-day variability, and the RSDs (%) of the peak areas were 1.82% and 0.88%, respectively. The stability was good, with the RSD (%) of the peak area reaching 0.57% within 24 h, and the repeatability was acceptable, with the RSD (%) of the content reaching 1.13%. The accuracy of the method was verified by the sample recovery test. The results showed that the average recovery was 98–105%, and the RSD (%) was 1.22%. All the above results demonstrated that the alternative method would suffice for the quantification requirement of secologanic acid in LJF and LF.

### 2.8. Quantitative Analysis of Samples

**Figure 6:**
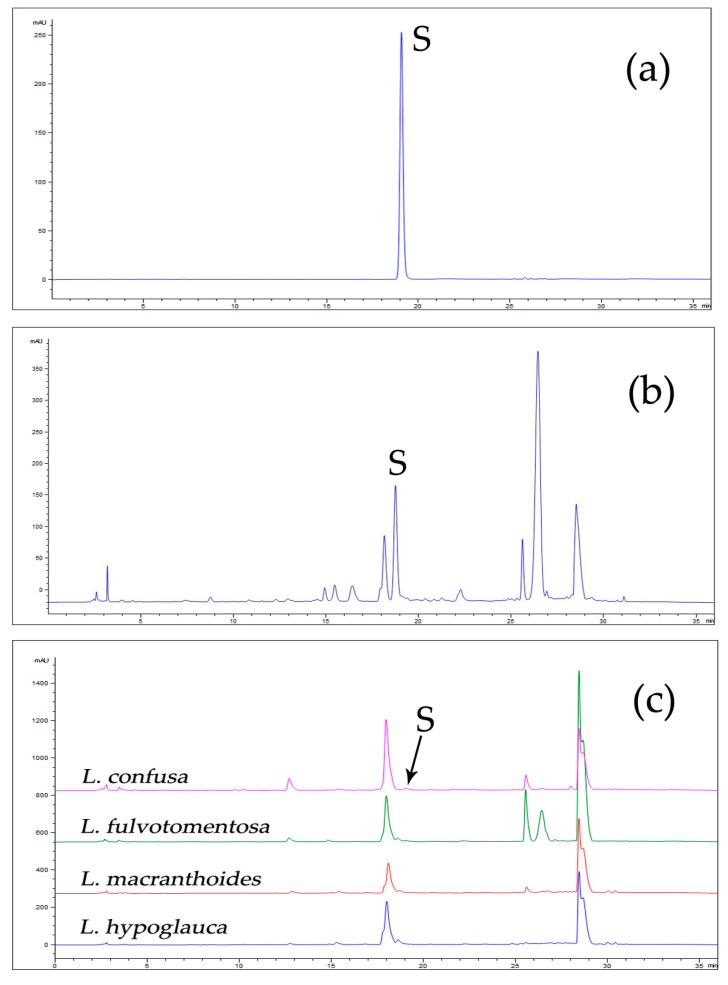
The HPLC chromatograms of standard (**a**) and samples. (**b**) LJF sample; (**c**) LF sample; S: secologanic acid. Column: An Agilent ZORBAX XDB C18 (4.6 × 250 mm, 5 μm) column; column temperature, 30 °C; mobile phases: 0.1% phosphoric acid solution (A) and acetonitrile (B), using a gradient elution of 5–15% B at 0–20 min, 15–23% B at 20–25 min, 23–95% B at 25–26 min, and 95% B at 26–30 min; flow rate: 1.0 mL/min; detection wavelength: 240 nm; injection volume: 10 µL.

The sample information is listed in Table 1 and Table 2. The validated analytical method was employed to assess the amount of secologanic acid in 34 batches of LJF and 38 batches of LF. The typical HPLC chromatography is shown in Figure 6, and the results for the amounts of secologanic acid are summarized in Table 1 and Table 2. The average amount of secologanic acid in 34 batches of LJF was 18.24 mg/g. The highest amount (23.3 mg/g) was found in HN-2-2 and the lowest (12.9 mg/g) was found in HB-3-1; however, the amount of secologanic acid in 38 batches of LF ranged from 0.2 mg/g to 7.2 mg/g, and the average was 1.76 mg/g.

### 2.9. Scatter Plot of the Samples

**Figure 7:**
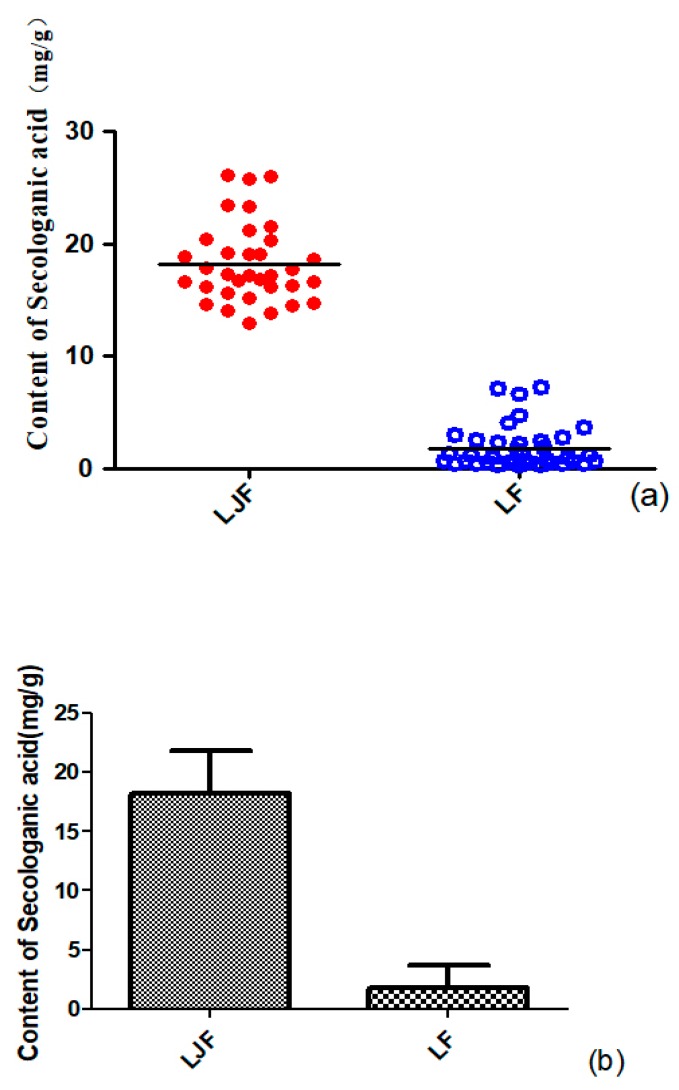
Scatter plot acquired from LJF and LF samples (**a**) and the contents of secologanic acid in LJF and LF (**b**) (*x*-axes show LJF and LF, the different sample; *y*-axes have the content of secologanic acid).

A scatter plot of all the samples was used to distinguish and assess the quality of LJF and LF. The measured amounts (mg/g) of secologanic acid in samples were set as variables. The scatter plot is displayed in Figure 7a. All the samples were divided into two clusters (LJF and LF), which indicated that the amounts of secologanic acid of LJF were quite different from LF.

### 2.10. Rank Sum Test of Samples

Because both groups of data (secologanic acid content of LJF and LF) did not conform to normal distribution, a rank sum test was used to verify the probability of the differences occurring, instead of a *t*-test, and the test type was Mann–Whitney. According to the results of the rank sum test, the sum of ranks of group 1 (LJF) was 1887.00, and the sum of ranks of group 2(LF) was 741.00, and the *p*-value was 0.000. Therefore, significant differences were found between LJF and LF in the amount of secologanic acid. As shown in Figure 7b, the amount of secologanic acid was strikingly higher in LJF than in LF, so secologanic acid can be considered as one of the characteristic components for distinguishing LF from LJF.

## 3. Discussion

*Lonicerae japonicae* flos is an important traditional Chinese medicine with various medicinal and edible benefits. However, in recent years, LJF has often been substituted for or adulterated with LF. In this study, one of the differential components in the HPLC fingerprinting of LJF and LF was prepared using column chromatography and pre-HPLC and was identified as secologanic acid. The amount of secologanic acid in 34 batches of LJF and 38 batches of LF were determined; the results showed that the amount of secologanic acid in LJF was significantly higher than that in LF. Therefore, secologanic acid can be used as one of the characteristic components for identifying LJF and LF.

A lot of diverse compounds, including phenolic acids, flavonoids, and iridoid glycosides, have been isolated from LJF samples [6]. Iridoids, are one of the most abundant compounds in LJF, of which 83 kinds have been identified to date, including iridoid glycosides, secoiridoid glycosides, and nitrogen-containing iridoid glycosides [6]. Traditionally, LJF was mostly used in water decoction. Thus, it was inferred that the water-soluble iridoids played vital roles in the efficacy of LJF. In the past few decades, iridoids have not been given enough attention in relation to the quality evaluation of LJF; the reason may be that some of the quantitative compounds, such as loganin, sweroside, and centauroside, are relatively low in content [16,17,24]. In this study, the average amount of secologanic acid in 34 batches of LJF was 18.24 mg/g, which is similar to the amount of chlorogenic acid in LJF; thus, it could be used as representative of iridoids in LJF samples. In addition, it has been reported that secologanic acid is easily destroyed and generates its sulfonates in the sulfur fumigation process, which leads to the amount of secologanic acid in fumigated LJF being significantly lower than that in non-sulfur fumigated samples [22]. Therefore, secologanic acid should be taken as one of the marker compounds for the quality evaluation of LJF, which could benefit the identification of LJF and LF, as well as the differentiation of sulfur-fumigated and non-sulfur fumigated LJF samples.

## 4. Materials and Methods

### 4.1. Plant Materials, Chemicals, and Reagents

The 34 batches of LJF and 38 batches of LF were taken from different habitats and commercial herbs. Detailed information on these samples is listed in Table 1 and Table 2. The botanical origins of materials were authenticated by one of the authors, Professor Yongqing Zhang. Voucher specimens were deposited at the Herbarium in Shandong University of Traditional Chinese Medicine.

Secologanic acid and chlorogenic acid were purchased from Shanghai Standard Biotechnology Co. Ltd. (Shanghai, China), and the purities of the two standard components were determined to be more than 98% by HPLC analysis. Further, HPLC-grade acetonitrile and methanol were purchased from Thermo Scientific (Thermo Scientific, MA, USA). Analytical grade phosphoric acid, acetic acid, ethanol, and methanol were purchased from Tianjin Kemiou Reagent Co. Ltd. (Tianjin, China). Distilled water was obtained from a Milli-Q purification system (Billerica, MA, USA).

### 4.2. Instruments

An Agilent 1260 liquid chromatography system (Agilent Technologies, Waldbronn, Germany), equipped with a quaternary solvent delivery system, an online degasser, an automatic sampler, a diode array detector, and a column oven, was employed. A semi-preparative HPLC (Shimadzu Corp., Kyoto, Japan) system, consisting of a LC-6AD dual ultra-high-pressure gradient pump and an SPD-20A detector was used. An UltiMate 3000 UPLC system coupled to a Q Exactive Hybrid Quadrupole-Orbitrap Mass Spectrometer (Thermo Scientific, MA, USA) was used.

### 4.3. HPLC Fingerprint Chromatogram Comparison of LJF and LF

Accurately, the amount of chlorogenic acid was weighed and dissolved in 50% methanol to get a final concentration of 0.379 mg·mL^−1^. The fine powders (0.5 g) of each sample, including LJF and LF, were accurately weighed and extracted by ultrasonication (250 W, 100 Hz) in 50mL of 50% methanol for 60 min. After being cooled to room temperature, the mixtures were weighed again, and the weights lost were replenished with 50% methanol, mixed well, and then filtered. Then, each methanol solution was filtered through a 0.45 μm membrane prior to HPLC.

An Agilent ZORBAX XDB C_18_ (4.6 × 250 mm, 5 μm) column was employed, and the column temperature was maintained at 30 °C. The mobile phase consisted of 0.1% phosphoric acid solution (A) and acetonitrile (B), using a gradient elution of 5–15% B at 0–20 min, 15–55% B at 20–45 min, and 55–100% B at 45–60 min. The flow rate was 1.0 mL/min, and an aliquot of 10 µL was injected. The detection wavelength was set at 254 nm.

### 4.4. Preparation of Crude Extracts Containing Compound S

The LJF sample (800 g) was powdered and sieved through a number 2 mesh and extracted with heating reflux for 2 h, 10, 10, and 8 times with 50% ethanol. The extraction was filtered, merged, and then concentrated to an appropriate volume. The concentrate was then separated over a D101 macroporous resin column and eluted with H_2_O and ethanol of different concentrations (10%, 20%, 30%, 40%, 50%, and 60%). The eluent was collected and analyzed using HPLC by the chromatographic conditions of HPLC fingerprinting. The peak area of compound S was recorded. The fraction with a higher content of compound S and fewer impurities was chosen as the crude extract for preparing compound S.

### 4.5. Separation and Purification of Compound S

The 30% ethanol eluent was separated over a Sephadex LH-20 column and eluted with 10% MeOH. Each eluent was collected and analyzed using HPLC. For shortening the analysis time, the gradient eluent procedure was slightly changed on the basis of the chromatographic conditions of HPLC fingerprinting, and the other conditions remained unchanged. The specific procedure was as follows: 0.1% phosphoric acid solution was used as mobile phase A and acetonitrile as mobile phase B, with 5–15% B at 0–20 min, 15–100% B at 20–25 min, and 100% B at 30 min.

The eluents containing compound S were mixed and dried after their solvent was recovered. Then, the dried concentrate was dissolved in 20% ethanol and further purified using the Shimadzu semi-preparative HPLC system. The mobile phase consisted of methanol (A) and pure water (B), using the gradient eluents of 85–45% B at 0–25 min, and 45–5% B at 25.1–40 min; and the detection wavelength was set at 240 nm; the flow rate was 10.0 mL/min, and 1 mL of each sample was injected. The eluents were collected and dried, and their structure was identified.

### 4.6. Identification of Compound S

To identify the structure of compound S, LC-MS analysis was performed using an UltiMate 3000 UPLC system (Thermo Scientific, USA) coupled to a Q Exactive Hybrid Quadrupole-Orbitrap Mass Spectrometer (Thermo Scientific, USA). The HPLC conditions were as follows: a Waters C_18_ column (2.1 × 100 mm, 1.7 µm) was used; the column temperature was set at 30 °C; the mobile phase consisted of 0.05% HCOOH (A) and acetonitrile (B), and the linear gradient was as follows: 5–15% B at 0–20 min, 15–55% B at 20–45 min, and 55–100% B at 45–60 min; the flow rate was 0.3 mL/min, and the injection volume was 0.1 µL. The parameters for LC-MS analysis were as follows: dry temperature, 350 °C; the velocities of sheath gas and auxiliary gas were 30 and 10, respectively; the capillary voltage was 3500 V; full-scan mass spectra were acquired in the positive and negative ionization modes over the range *m/z* 100–1200.

### 4.7. Stability of Secologanic Acid

The stability of secologanic acid in H_2_O, 20% ethanol, 50% ethanol, and 50% methanol was tested. About 0.25 g powder of the LJF sample was accurately weighed and extracted with 25 mL of H_2_O, 20% ethanol, 50% ethanol, and 50% methanol, separately. The extraction method was in accordance with “item 4.8.1,” and the solution was prepared in triplicate and then analyzed by the chromatography condition of “item 4.8.2.” The sample solution was injected every 3 h within 27 h, and the peak area was recorded and the RSD (%) was calculated.

### 4.8. Determination of Secologanic Acid in LJF and LF

#### 4.8.1. Preparation of Standard Solution and Sample Solution

The secologanic acid was weighed accurately and dissolved in 20% ethanol to concentrations of 1.132 mg/mL and 0.233 mg/mL.

The fine powders (0.5 g) of each sample, including LJF and LF, were accurately weighed and ultrasonically extracted (300 W, 40 kHz) in 25 mL of 20% EtOH for 30 min. After being cooled to room temperature, the lost weight of each was replenished with 20% ethanol, and then the solutions were well mixed and filtered through 0.45 μm membranes.

#### 4.8.2. Chromatographic Conditions

An Agilent ZORBAX XDB C_18_ (4.6 × 250 mm, 5 μm) column was employed, and the column temperature was maintained at 30 °C. The mobile phase consisted of 0.1% phosphoric acid solution (A) and acetonitrile (B) using a gradient elution of 5–15% B at 0–20 min, 15–23% B at 20–25 min, 23–95% B at 25–26 min, and 95% B at 26–30 min. The flow rate was 1.0 mL/min, and the injection volume was 10 µL. The detection wavelength was set at 240 nm.

#### 4.8.3. Validation of the Quantitative Method

An appropriate amount of secologanic acid was taken and diluted to a series of concentrations of 2.264, 5.66, 11.32, 56.6, 226.4, and 566 µg/mL with 20% EtOH. The solution was then analyzed using a chromatographic method. The calibration curves were plotted by peak area (Y) versus concentration. The detection limit was the concentration of a standard solution with a signal-to-noise ratio (S/N) of 3 (LOD), and the quantitative limit was the concentration of standard solution with an S/N of 10 (LOQ).

Accuracy was evaluated with the standard solution for six consecutive times. The stability test was performed with the same sample solution at 0, 2, 4, 8, 16, and 24 h. The RSD of the peak area was taken as a measure of precision and stability. The repeatability was determined with the six solutions prepared in parallel from the same sample, and the RSD of secologanic acid content was calculated.

A recovery test was used to evaluate the accuracy of the developed assay. Precise amounts of secologanic acid were added to approximately 0.25 g of sample powder (SD-1-1), which was then extracted and analyzed as described above. Each sample was analyzed in triplicate. The average percentage recoveries were evaluated by calculating the ratio of detected amount versus added amount.

### 4.9. Multiple Statistical Analysis

In order to observe the classification and assess the variation of LJF and LF, the scatter plot of all the samples including LJF and LF was made according to the amount of secologanic acid using GraphPad Prism 5 software. The rank sum test was utilized to confirm that there were differences in the amount of secologanic acid between LJF and LF (SPSS 16.0 for Windows, IBM, Armonk, NY, USA).

## 5. Conclusions

In this study, secologanic acid, a kind of iridoid which can be used to distinguish LJF from LF, was prepared and purified using column chromatography combined with pre-HPLC. The stability of secologanic acid in different solvents, including H_2_O, 20% ethanol, 50% ethanol, and 50% methanol, was studied for selecting the appropriate extraction solvent, and the results showed that secologanic acid could remain stable within 27 h when the sample of LJF was extracted with 20% ethanol. To establish the method of determining the secologanic acid content, the extraction conditions, including extraction time, solvent volume, extraction methods (ultrasonication or hot reflux), and the chromatographic conditions, were optimized. Then, the method for determining the amount of secologanic acid in LJF and LF was set up using HPLC. The results showed that the amount of secologanic acid was strikingly higher in LJF than in LF; therefore, it could be used for the identification of LJF from LF.

This was the first time that the differential component in the HPLC fingerprinting of LJF and LF was selected as one of marker components for the quality evaluation of LJF. Our study not only established a rapid, simple, sensitive, and practical method for distinguishing LJF from LF, but also provides a reference for discovering the characteristic components of other herb-medicines that are susceptible to adulteration.

## Figures and Tables

**Table 1:** Contents of secologanic acid in samples of LJF (*n* = 3, $\bar{x}$ ± SD).

Number	Batch Number	Producing Area	Content of Secologanic Acid (mg/g)
1	HN-1-1	Fengqiu, HeNan; cultivated	16.06 ± 0.48
2	HN-1-2	Fengqiu, HeNan; cultivated	16.61 ± 0.42
3	HN-1-3	Fengqiu, HeNan; cultivated	16.68 ± 0.52
4	HN-1-4	Fengqiu, HeNan; cultivated	17.23 ± 0.51
5	HN-2-1	Fengqiu, HeNan; cultivated	15.56 ± 0.47
6	HN-2-2	Fengqiu, HeNan; cultivated	23.29 ± 0.63
7	HN-2-3	Fengqiu, HeNan; cultivated	17.81 ± 0.53
8	HN-3-1	Fengqiu, HeNan; cultivated	21.53 ± 0.61
9	HN-3-2	Fengqiu, HeNan; cultivated	16.61 ± 0.50
10	HN-3-3	Fengqiu, HeNan; cultivated	21.2 ± 0.64
11	HN-4-1	Fengqiu, HeNan; cultivated	14.48 ± 0.44
12	HN-4-2	Fengqiu, HeNan; cultivated	14.7 ± 0.45
13	HN-4-3	Fengqiu, HeNan; cultivated	14.62 ± 0.45
14	SD-1-1	Pingyi, ShanDong; cultivated	19.23 ± 0.52
15	SD-1-2	Pingyi, ShanDong; cultivated	17.3 ± 0.54
16	SD-1-3	Pingyi, ShanDong; cultivated	14.03 ± 0.46
17	SD-1-4	Pingyi, ShanDong; cultivated	17.15 ± 0.53
18	SD-2-1	Pingyi, ShanDong; cultivated	26.01 ± 0.69
19	SD-2-2	Pingyi, ShanDong; cultivated	26.09 ± 0.61
20	SD-2-3	Pingyi, ShanDong; cultivated	16.24 ± 0.50
21	SD-3-1	Pingyi, ShanDong; cultivated	18.98 ± 0.49
22	SD-3-2	Pingyi, ShanDong; cultivated	16.1 ± 0.48
23	SD-3-3	Pingyi, ShanDong; cultivated	19.12 ± 0.53
24	SD-4-1	Pingyi, ShanDong; cultivated	13.85 ± 0.45
25	SD-4-2	Pingyi, ShanDong; cultivated	23.38 ± 0.54
26	SD-4-3	Pingyi, ShanDong; cultivated	18.63 ± 0.51
27	HB-1-1	Julu, HeBei; cultivated	25.79 ± 0.60
28	HB-2-1	Julu, HeBei; cultivated	20.32 ± 0.52
29	HB-2-2	Julu, HeBei; cultivated	18.94 ± 0.49
30	HB-3-1	Julu, HeBei; cultivated	12.94 ± 0.42
31	HB-3-2	Julu, HeBei; cultivated	15.11 ± 0.47
32	HB-3-3	Julu, HeBei; cultivated	17.67 ± 0.43
33	HB-4-1	Julu, HeBei; cultivated	20.38 ± 0.58
34	HB-4-3	Julu, HeBei; cultivated	16.77 ± 0.49

**Table 2:** Contents of secologanic acid in samples of LF (*n* = 3, $\bar{x}$ ± SD).

Number	Batch Number	Producing Area or Origin	Species	Content of Secologanic Acid (mg/g)
1	LF-M-1	Longhui, Hunan; cultivated	*L. macranthoides*	0.34 ± 0.02
2	LF-M-2	Longhui, Hunan; cultivated	*L. macranthoides*	0.39 ± 0.02
3	LF-M-3	Longhui, Hunan; cultivated	*L. macranthoides*	0.87 ± 0.04
4	LF-M-4	Guizhou; cultivated	*L. macranthoides*	0.57 ± 0.03
5	LF-M-5	Guizhou; cultivated	*L. macranthoides*	0.47 ± 0.02
6	LF-M-6	Guizhou; cultivated	*L. macranthoides*	0.61 ± 0.03
7	LF-M-7	Guizhou; commerical	*L. macranthoides*	0.43 ± 0.02
8	LF-M-8	Guizhou; commerical	*L. macranthoides*	0.57 ± 0.03
9	LF-M-9	Guizhou; commerical	*L. macranthoides*	2.24 ± 0.09
10	LF-M-10	Guizhou; commerical	*L. macranthoides*	0.67 ± 0.03
11	LF-M-11	Guizhou; commerical	*L. macranthoides*	0.59 ± 0.03
12	LF-M-12	Guizhou; commerical	*L. macranthoides*	0.64 ± 0.03
13	LF-F-1	Guizhou; cultivated	*L. fulvotomentosa*	3.62 ± 0.14
14	LF-F-2	Guizhou; cultivated	*L. fulvotomentosa*	2.67 ± 0.11
15	LF-F-3	Qin Zhou, Guangxi; cultivated	*L. fulvotomentosa*	0.89 ± 0.05
16	LF-H-1	Yizhou, Guangxi; cultivated	*L. hypoglauca*	0.52 ± 0.03
17	LF-H-2	Nanning, Guangxi; cultivated	*L. hypoglauca*	1.10 ± 0.06
18	LF-C-1	Medicinal botanical garden, Guangxi university of traditional chinese medicine; cultivated	*L. confuse*	7.08 ± 0.26
19	LF-1	Nanjiang, Si Chun; commerical	Mixed species ^#^	1.85 ± 0.08
20	LF-2	Chong qing; commerical	Mixed species ^#^	0.31 ± 0.02
21	LF-3	Chongqing; commerical	Mixed species ^#^	0.64 ± 0.03
22	LF-4	Bozhou, an hui; commerical	Mixed species ^#^	0.40 ± 0.02
23	LF-5	Wenshan, Yunnan; commerical	Mixed species ^#^	2.54 ± 0.10
24	LF-6	Dafang, Guizhou; commerical	Mixed species ^#^	0.43 ± 0.02
25	LF-7	Guizhou; wild	Mixed species *	2.30 ± 0.09
26	LF-8	Guizhou; wild	Mixed species *	4.67 ± 0.17
27	LF-9	Guizhou; wild	Mixed species *	7.19 ± 0.24
28	LF-10	Guizhou; wild	Mixed species *	6.62 ± 0.21
29	LF-11	Guizhou; wild	Mixed species *	4.01 ± 0.15
30	LF-12	Guizhou; wild	Mixed species *	2.85 ± 0.12
31	LF-13	Guizhou; wild	Mixed species *	2.40 ± 0.09
32	LF-14	Jingxi, Guangxi; commercial	Mixed species ^#^	1.29 ± 0.05
33	LF-15	Xinzhou, Guangxi; commercial	Mixed species ^#^	1.07 ± 0.05
34	LF-16	Guilin, Guangxi; commercial	Mixed species ^#^	0.53 ± 0.02
35	LF-17	Guanyang, Guangxi; commercial	Mixed species ^#^	0.20 ± 0.01
36	LF-18	Hechi, Guangxi; commercial	Mixed species ^#^	1.57 ± 0.06
37	LF-19	Nanning, Guangxi; commercial	Mixed species ^#^	1.34 ± 0.05
38	LF-20	Baise, Guangxi; commercial	Mixed species ^#^	0.59 ± 0.03

^#^ purchased from dealers of LF in the market, and mixtures of two or more of the four species of LF; * purchased from a herbalist who collected the LF samples on a mountain, and mixtures of two or more of the four species of LF.
